# Patient Innovation in Investigating the Effects of Environmental Pollution in Schizophrenia: Case Report of Digital Phenotyping Beyond Apps

**DOI:** 10.2196/19778

**Published:** 2020-08-03

**Authors:** Aditya Vaidyam, Spencer Roux, John Torous

**Affiliations:** 1 Division of Digital Psychiatry Beth Isreal Deaconess Medical Center Harvard Medical School Boston, MA United States

**Keywords:** digital mental health, mHealth, apps, serious mental illness, schizophrenia, psychiatry, digital phenotyping

## Abstract

This patient perspective highlights the role of patients in the innovation and codesign of digital mental health technology. Though digital mental health apps have evolved and become highly functional, many still act as data collection silos without adequate support for patients to understand and investigate potentially meaningful inferences in their own data. Few digital health platforms respect the patient’s agency and curiosity, allowing the individual to wear the hat of researcher and data scientist and share their experiences and insight with their clinicians. This case is cowritten with an individual with lived experiences of schizophrenia who has decided to openly share their name and experiences to share with others the methods and results of their curiosity and encourage and inspire others to follow their curiosity as well.

## Introduction

Digital health software, including apps, are designed to serve people and help improve clinical outcomes. Although apps have become more functional and able to capture both surveys and sensor information (ie, digital phenotyping [1]), technical limitations in smartphone software and hardware limit potential data capture. Highlighting the potential of health software beyond smartphones (apps), this case report demonstrates the potential for such software to transition between different devices and hardware toward better serving people. Building off a prior case report where an individual started with an app to track symptoms but found a tally counter more useful [2], here we discuss a case where an app was used at first, followed by a transition to a novel device that proved to be a more comprehensive solution.

## Brief Case

Spencer Roux (henceforth referred to as SR) is a male in his thirties who was diagnosed with schizophrenia in 2013. His symptoms are currently well managed with exercise, lifestyle interventions, and medications. Seeking to constantly improve his condition and curious about the impact of various environmental aspects on his mental health, SR sought to quantify such environmental aspects. SR also considered tracking noise level, temperature, and ambient lighting, the latter serving as a proxy for sunlight.

Mental health concerns related to environmental variations, including global warming, climate change, and pollution are rising while pollution itself is a known trigger for brain inflammation [3-5], which may be related to the onset and exacerbation of numerous illnesses. The strongest available evidence is for the relationship between pollution and depression [6] but emerging evidence indicates that ambient pollution could be associated with hospitalization in illnesses such as schizophrenia [7,8]. However, tracking environmental conditions such as pollution is not possible today through the use of common smartphone apps, as no major smartphone devices support indoor precision air quality sensors. Although it is possible to purchase such sensors individually, there is no off-the-shelf indoor precision air quality sensor that also tracks mental health available on the market today.

Though he had no prior knowledge of this literature, SR wanted to assess the relationship between air quality and the number of auditory hallucinations he experienced day-to-day. As his symptoms are well controlled, he believed this was a practical time to learn more about environmental factors that may be related to his symptoms. Previously, SR used a tally counter device to identify a relationship between his auditory hallucination count and the number of times he responded to his hallucinations [2], and here sought to capture new data. As with the tally counter, he wanted to track his symptoms in a manner that was inconspicuous and easy to engage with.

**Figure 1:**
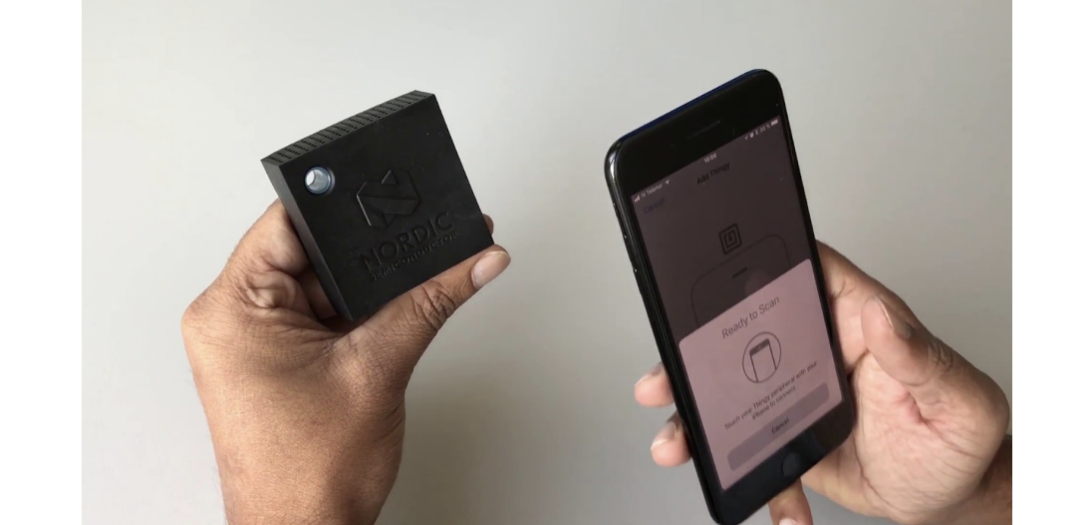
The size of a Nordic Thingy shown in comparison to a smartphone device. The primary input method is the surface containing the Nordic Semiconductors logo, which acts as a pressure-insensitive button pad.

The Nordic Thingy prototyping platform (Figure 1; Nordic Semiconductor) was temporarily provided to SR at no cost as it would be suitable for his needs and is compatible with the LAMP platform, which enables visualization and statistical inference. The LAMP platform has been used in prior clinical cases for similar purposes [9], using only sensors available on commercial smartphone devices. The Nordic Thingy prototyping platform has numerous sensors not available in a smartphone that are able to achieve a high fidelity of measurement recording. The device was preloaded with a long-term evolution (LTE) data plan. The sensors include the following: LTE, Bluetooth, Wi-Fi, near-field communication (NFC), button, temperature, humidity, air quality, air pressure, ambient color, ambient light, high-g accelerometer, low-power accelerometer, global position system (GPS), digital microphone, radio frequency (RF) antenna, barometer, altitude, 9-axis motion tracker. It also has a multicolor light emitting diode (LED) and digital buzzer (a low-quality speaker) for responding to the user if necessary.

Due to its literal “black box” nature, it was not distracting to use, and was convenient and easy to manage. Noting its primary purpose as a prototyping system, it was purchased from a Nordic Semiconductors distributor for US $120. As the Nordic Thingy can capture data but does not offer data management, integration, or visualization, the LAMP platform was then installed to enable digital mental health–related functionality [10,11]. The Nordic Thingy was charged roughly alongside SR's smartphone and captured or measured the environment of SR's home throughout the day, for most of which SR was present. The button press was used to record auditory hallucinations, similar to the tally counter SR had used previously.

**Figure 2:**
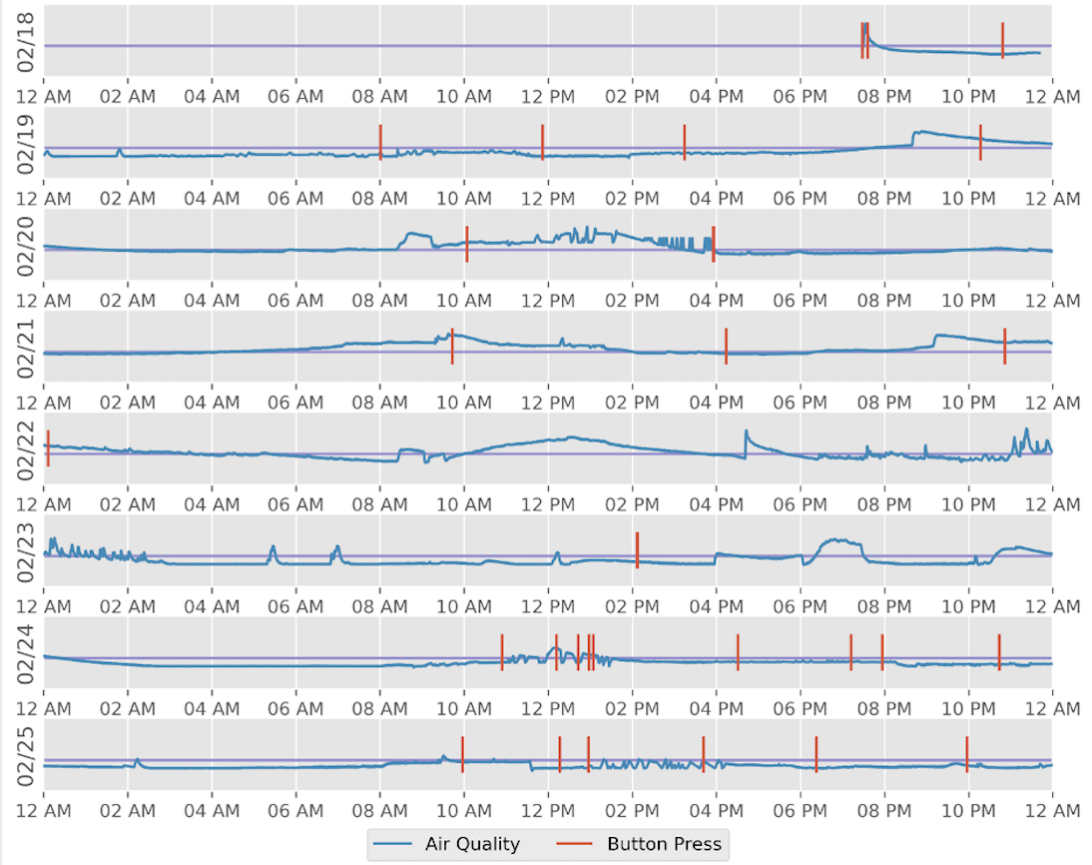
SR's experimental data. The red lines indicate a button press, the blue line indicates indoor air quality, and the purple line indicates the threshold for indoor air quality >100; higher values indicate poorer air quality.

SR independently collected over 200,000 data points via the Nordic Thingy in the span of seven days, producing about 65 MB of uncompressed data. The humidity, temperature, air pressure, and air quality data appeared as sensor data in the LAMP platform, and the button presses appeared as active data in the form of responses to a survey with a single yes or no question. With the assistance of the LAMP programming interfaces, the data were plotted and are shown in Figure 2.

Though his findings showed no significant relationship between indoor air quality and auditory hallucinations (McNemar chi-squared test with continuity correction = 3179.4, *df*=1, *P*<.001), SR was able to devise a hypothesis, perform an experiment, analyze the data, and produce a tangible outcome within the span of 1 week. The installation of the LAMP platform on a non–smartphone device proved useful in yielding high-quality data suitable for actionable digital phenotyping as well as data visualization.

## Brief Discussion

As a single case report, SR's example highlights the potential for innovation in and codesign of digital mental health technology by those with lived experiences of serious mental illness. As technology and sensors evolve, it is possible to collect novel data streams like pollution on a highly personalized scale. Understanding the role of digital mental health technology beyond just apps and ensuring it is flexible enough to move between devices and contexts ensures that both patients and clinicians can take full advantage of these advances in technology. The design and evolution of digital mental health tools and platforms will likely rapidly evolve toward devices similar to the one used in this case report, and must involve individuals such as SR, whose lived experiences offer insight into both the problems and solutions encountered when building and using such systems.

As novel sensors like those used in this case report continue to add new accessible data, it is also critical for the field to understand their clinical uses, personal meaning, and therapeutic targets. Although it is easy to use such data in machine learning algorithms or artificial intelligence systems, starting with patient-centric clinical use cases can minimize bias, increase utility, and accelerate clinical translation. From an experimental methods standpoint, it is also important to consider privacy, safety, and ethics when developing individual study protocols, ensuring that all parties involved obtain recorded consent and respect the wishes of other parties. Furthermore, as seen in this case example, there was actually no need for advanced analytical methods beyond simple visualization and a statistical test, highlighting how the right data used in the right clinical case can itself be definitive.

As SR's case yet again shows, a smartphone app may not always be the solution. Although smartphones and wearables are considered highly accessible and usable devices that are practically always within reach, the potential of other digital hardware should not be ignored. The software can be the same, as demonstrated by the use of the LAMP platform on a non–smartphone device. Focusing less on the device and more on how the software serves unique clinical purposes offers a useful reminder toward focusing on fluid and interoperable technology that is not tied to any one platform or device. SR's case shows the merits of using non–smartphone devices for their specialized sensors and unobtrusive behavior.

Through a unique experimental journey, SR's case presents a clear message to other curious and innovative individuals with lived experiences, as well as creators and implementors of digital mental health technologies. Clinicians may also choose to actively take part or passively encourage innovation, lending clinical context and insight in either case. Through creativity, innovation, and in small part the accessibility and actionability of data, individuals given the right tools can understand and impact their own mental health.

## Abbreviations

GPS: global positioning system
LED: light emitting diode
LTE: long-term evolution
NFC: near-field communication
RF: radio frequency
